# Identification of a novel *NRF1*::*PDGFRA* fusion in myeloid/lymphoid neoplasms with eosinophilia and tyrosine kinase gene fusions

**DOI:** 10.3389/fonc.2025.1552928

**Published:** 2025-03-25

**Authors:** Jialong Liu, Yaqing Feng, Yanfang Zhang, Yingnan Xiao, Xi Liu, Tingting Xiao, Junyan Zou, Kai Fan, Lisha Lu, Xiaoxia Yang, Jinying Gong

**Affiliations:** ^1^ Hematology Pathology Diagnostic Center, TianjinUnion Precision Medical Diagnostics Co. Ltd, Tianjin, China; ^2^ Department of Hematology, The Third People’s Hospital of Datong City, Datong, Shanxi, China; ^3^ State Key Laboratory of Experimental Hematology, National Clinical Research Center for Blood Diseases, Haihe Laboratory of Cell Ecosystem, Institute of Hematology and Blood Diseases Hospital, Chinese Academy of Medical Sciences and Peking Union Medical College, Tianjin, China

**Keywords:** eosinophilia, PDGFRA rearrangement, NRF1, MLN-TK, gene fusion, imatinib

## Abstract

A novel fusion gene *NRF1::PDGFRA* was identified in a patient with myeloid/lymphoid neoplasms with eosinophilia and tyrosine kinase gene fusions (MLN-TK), harboring the chromosome abnormality t(4;7)(q12;q32). This represents the first reported case of the *NRF1*::*PDGFRA* fusion gene, and the ninth *PDGFRA*-associated fusion gene identified in MLN-TK. The fusion event led to the constitutive activation of the PDGFRA kinase, resulting in uncontrolled eosinophil proliferation and potentially contributing to the occurrence of cerebral infarction. Our study indicates treatment with low-dose imatinib effectively alleviates the symptoms associated with *NRF1::PDGFRA* gene fusion.

## Introduction

The World Health Organization (WHO) 2022 classification and the International Consensus Classification of Myeloid and Lymphoid Neoplasms (ICC-MLN) define a distinct subcategory of myeloid neoplasms as “myeloid/lymphoid neoplasms with eosinophilia and tyrosine kinase gene fusions” (MLN-TK), which are driven by rearrangements/fusion genes involving *PDGFRA*, *PDGFRB*, *FGFR1*, *JAK2*, *ABL1*, or *FLT3* (1–3). Notably, myeloid and lymphoid neoplasms with eosinophilia and *PDGFRA* rearrangements are recognized as a distinct entity within the section of MLN-TK (1, 4). At the time of writing, all fusion partners of *PDGFRA* that have been described in MLN-TK, including *FIP1L1*, *BCR*, *ETV6*, *KIF5B*, *CDK5RAP2*, *STRN*, *TNKS2*, *FOXP1*, and *AKAP9* (4–13). The *FIP1L1* gene is the most frequent fusion partner, followed by the *BCR* gene, with other partner genes being infrequent. In this study, we describe the identification of *NRF1* as a novel and rare fusion partner of *PDGFRA* in an adult patient with MLN-TK, and provide a detailed account of the patient’s clinical course following treatment with low-dose imatinib.

## Methods

### Chromosomal analysis

Chromosomal analysis was performed by examining short-term cultures of bone marrow (BM) according to standard conventional cytogenetic protocols. Fresh bone marrow was collected from each patient and cultured for 24 h (in RPMI 1640 medium, 20% calf serum) without any growth factors. A methanol–glacial acetic acid fixation method was used for obtaining metaphase cells, with G-banding and R-banding performed. Analysis was performed using an Ikaros automated scanning system (Metasystems, Germany). At least 20 cells in metaphase were analyzed in each case. Karyotype descriptions are based on the International System for Human Cytogenomic Nomenclature (ISCN 2020).

### Fluorescence *in situ* hybridization

BM of this patient was collected and processed following standard cytogenetic protocols. For interphase fluorescence *in situ* hybridization (FISH), the slides containing fixed cells were incubated in 2× SSC at 37°C for 30 minutes, rinsed with deionized water, and subsequently dehydrated through graded ethanol series (70%, 85%, and 100%). After air drying, slides were denatured at 83°C for 5 minutes in the water bath, hybridized with *PDGFRA* Tricolor Rearrangement Probe (HealthCare, China) at 37°C for 16 hours using the hybridizer (Abbott, USA), stained with DAPI, and analyzed under the fluorescence microscope (Zeiss, Germany). For metaphase FISH, the labeled slides from chromosome analysis should undergo de-oiling and destaining before incubation in 2× SSC buffer.

### RNA sequencing

Total RNA was extracted from diagnostic BM using TRIzol, following the manufacturer’s protocol. 500-1000 ng of RNA was Poly(A)-based mRNA enrichment using VAHTS mRNA Capture Beads 2.0 (Vazyme, China). The preparation of cDNA library using VAHTS Universal V8 RNA-seq Library Prep Kit for Illumina (Vazyme, China). Finally, Illumina Novaseq 6000 sequencing platform was used for library sequencing. Raw FASTQ data were quality-checked with Fastp, and fusion gene candidates were identified using Arriba (v2.2.0). Hot gene pairs were reviewed with an in-house database and manually verified in Integrative Genomics Viewer (v2.16.2).

### TaqMan RT-qPCR

Total RNA was reverse transcribed into cDNA using the High-Capacity cDNA Reverse Transcription Kit (Thermo Fisher, USA) according to the manufacturer’s instructions. RT-qPCR was performed in a 96-well plate (20 µL/well) containing TaqMan gene Expression Master Mix, primers (500 nM), probe (250 nM), and cDNA template. The primers and probe sequences are provided in **Supplementary Figure 1E**. Thermal cycling was as follows: 50°C for 2 min, 95°C for 10 min, followed by 45 cycles of 95°C for 15 sec and 60°C for 1 min. c*ABL* was used as a housekeeping gene to confirm the amplifiability and quality of the cDNA.

## Results

A 27-year-old male patient with a history of recurrent pruritus and irritative cough was admitted to the hospital complaining about the persistent eosinophilia and recurrent episodes of dizziness. Six months earlier, he had experienced a sudden onset of left-sided limb weakness and dysarthria. At that time, Cranial computed tomography (CT) demonstrated a wedge-shaped hyperintense lesion at the right frontotemporal-parietal junction, indicative of a cerebral infarction. His peripheral blood exhibited a mildly elevated white blood cell count (18.42×10^9^/L) with marked eosinophilia (43.2%), and thrombocytopenia (73×10^9^/L). No hepatosplenomegaly was detected upon physical examination. Cerebrospinal fluid was normal.

Bone marrow (BM) morphology showed marked granulocytic hyperplasia with an increase in cytoplasmic granules within granulocytes and a heightened proportion of eosinophils (**Supplementary Figure 1A**). BM biopsy indicated extremely active marrow proliferation (>90%) with an increased proportion of granulocytes, and noticeable eosinophil and megakaryocyte proliferation. Flow cytometry revealed a low proportion of myeloid blasts with no significant phenotypic abnormalities, and an increased proportion of eosinophils (**Supplementary Figure 1B**).

**Figure 1:**
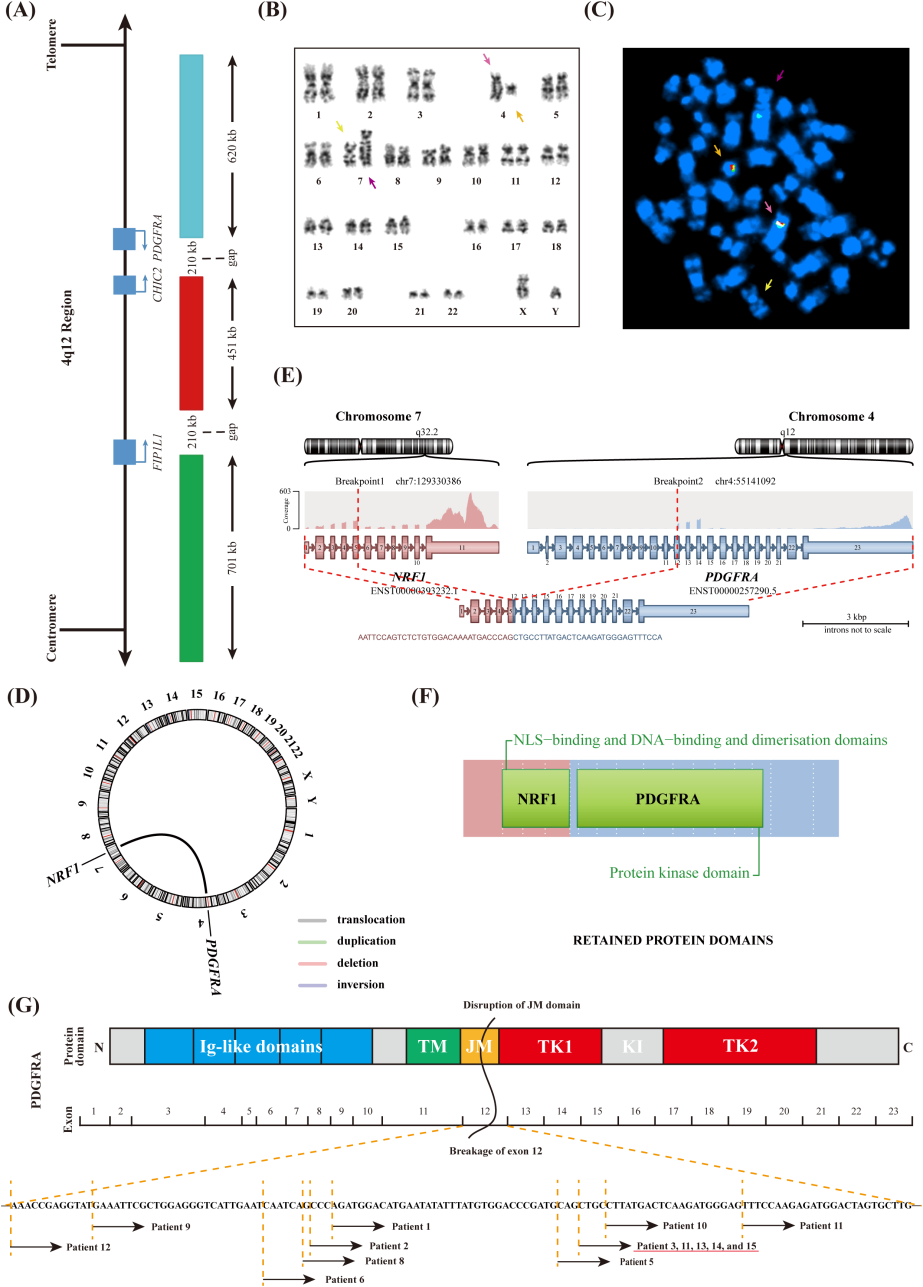
Molecular Genetics analyses of BM cells. **(A)** Schematic representation of the 4q12 region indicating relevant genes and the *PDGFRA* Tricolor Rearrangement Probe. **(B)** Karyotype showing abnormal chromosomes identified by G-banding. **(C)** Metaphase FISH hybridized with *PDGFRA* Tricolor Rearrangement Probe. **(D)** Identification of a novel *NRF1*::*PDGFRA* fusion gene by RNA sequencing. **(E)** Schematic representation of the recombination between the *NRF1* and *PDGFRA* genes. **(F)** The domain of the NRF1-PDGFRA fusion protein. **(G)** Schematic representation of the PDGFRA protein domains and the break positions within exon 12 of PDGFRA in MLN-TK patients.

Conventional chromosome analysis using G-banding and R-banding demonstrate a male karyotype with an apparently balanced t(4;7)(q12; q32) observed in 16/20 BM cells examined (**Figure 1B**, **Supplementary Figure 1C**). Interphase FISH analysis using the *PDGFRA* Tricolor Rearrangement Probe (**Figure 1A**) revealed a 71% positive signal (**Supplementary Figure 1D**). One overlapping green/orange/aqua signal on the normal chromosome 4 (pink arrows), one overlapping green/orange signal on the abnormal chromosome 7 (orange arrows), and one separate aqua signal on the abnormal chromosome 4 (purple arrows) were detected by metaphase FISH (**Figure 1C**). Normal chromosome 7 had no signal (yellow arrows).

The abnormal FISH signal in this case indicated that *PDGFRA* was cleaved and that the telomere proximal site of chromosome 4q was translocated to chromosome 7q, which had not been previously reported. Consequently, the RNA sequencing analysis was conducted to identify the unknown fusion partner, and the *NRF1* (Nuclear Respiratory Factor 1; Gene ID: 4899) was confirmed at the RNA level (**Figures 1D, E**). Quantitative analysis revealed that *NRF1*::*PDGFRA* transcript exhibited a higher expression level (210.72%). Based on the above results, we confirmed the in-frame fusion between the intact exon 5 of *NRF1* and the truncated exon 12 of *PDGFRA*. The resulting fusion protein comprised the nuclear localization signal (NLS)-binding and DNA-binding and dimerization domains of NRF1, along with the protein kinase domain of PDGFRA (**Figure 1F**).

The patient was diagnosed with MLN-TK and initiated treatment with imatinib at a daily dose of 100 mg. After three days, the patient’s pruritus significantly improved. After two weeks, the cough had fully resolved, and dizziness had largely subsided. Follow-up complete blood count results revealed a white blood cell count of 5.25×10^9^/L, an eosinophil percentage of 3.6%, and a platelet count of 172×10^9^/L, indicating the achievement of complete hematologic remission. After three months of treatment, both chromosome analysis and fluorescence *in situ* hybridization (FISH) analysis were negative, confirming the achievement of complete cytogenetic remission. Additionally, quantitative analysis of the *NRF1*::*PDGFRA* fusion gene demonstrated a substantial reduction from 210.72% to 0.11%, suggesting partial molecular remission. Magnetic resonance imaging (MRI) scans revealed a marked decrease in high signal intensity within the right frontal-temporal-parietal junction cortex compared to prior imaging. However, residual patchy high signal lesions persisted in the cortical region, accompanied by localized gliosis and mild atrophic changes (**Supplementary Figure 1F**). During the course of imatinib therapy, the patient experienced mild (grade 1) myalgia in the limbs, which spontaneously resolved after approximately one month, likely attributed to the imatinib treatment.

## Discussion

MLN-TK encompasses a wide range of histological types, including MPN, MDS, MDS/MPN, AML, and MPAL, as well as B or T lymphocytic leukemia/lymphoma (ALL). Extramedullary disease is common. Although eosinophilia is a frequent and significant feature, it may be absent in some cases (1). In the reported cohort of 135 MLN-TK patients, blast phase was primary in about 70% of patients, with a lower relative frequency of 16% in patients with *PDGFRA*/*PDGFRB* fusion genes, and only 6% had secondary blast phase after a median of 87 months, likely because >90% of patients achieved persistent complete hematologic, complete cytogenetic (*PDGFRB*), and complete molecular (*FIP1L1*::*PDGFRA*) remissions after treatment with imatinib (3, 11).

NRF1 functions as a transcription factor that activates the expression of some key metabolic genes regulating cellular growth and nuclear genes required for respiration, heme biosynthesis, and mitochondrial DNA transcription and replication (14). The fusion partners of *NRF1* that have been discovered through Next-Generation Sequencing (NGS) include *MTMR1*, *BRAF*, *STRIP2*, *PPP2R5A* and *MKLN1*, among others (15, 16). However, only the *NRF1*-*BRAF* fusion has been reported in two cases, associated with pleomorphic xanthoastrocytoma and urothelial carcinoma, with genomic breakpoints in *NRF1* occurred within exon 5 and exon 10, respectively (17, 18). Regrettably, the functional alterations resulting from *NRF1* rearrangements have scarcely been explored in the literature. Nevertheless, research has revealed that the knockdown of *NRF1* in rats adversely impacts mitochondrial biogenesis and function, and the knockout of *NRF1* in mice even resulting in embryonic lethality (19). The impact of *NRF1* rearrangements remains to be further studied.

*PDGFRA* rearrangements typically activate receptor tyrosine kinases (RTKs), initiating a cascade of aberrant signaling pathways, which ultimately result in uncontrolled cell proliferation and inhibition of apoptosis (20). As shown in **Figure 1G**, PDGFRA consists of five extracellular immunoglobulin-like (Ig-like) domains, a transmembrane (TM) domain, a juxtamembrane (JM) domain, and a bipartite tyrosine kinase catalytic domain (TK1 and TK2), which are separated by a kinase insertion region (KI) (21). An unusual phenomenon has been observed in which the breakpoint in *PDGFRA* always occurs within exon 12 (**Figure 1G**, **Table 1**). Previous studies have demonstrated that exon 12 of *PDGFRA* encodes a portion of the JM domain, which is known to exert an autoinhibitory function in other receptor tyrosine kinases. Disruptions to this domain, including missense mutations, in-frame insertions, or in-frame deletions, can result in the constitutive activation of the corresponding tyrosine kinase (22). For this phenomenon, we believe it is related to the special position of exon 12 that encodes the JM domain. If the breakage position is before exon 12, the complete JM domain will inhibit the activity of the RTKs. Conversely, if the break occurs after exon 12, the disrupted TK domain leads to RTKs inactivation. Both scenarios do not result in disease phenotypes and thus remain undetected. Similar to other fusion tyrosine kinases, NRF1-PDGFRA is also likely a constitutively active tyrosine kinase that can transform hematopoietic cells both *in vitro* and *in vivo*, consistent with mechanisms observed in previous studies, although further investigation is necessary to substantiate the mechanism.

**Table 1:** The fusion partners of *PDGFRA* in MLN-TK patients and their clinical treatment outcomes.

Patient	Age/sex	Fusion partner	Fusion exon	Additional genetic/molecular abnormalities	Main treatment modalities	Follow-up	Treatment outcomes	Reference
1	39/M	*FIP1L1* (4q24)	E8-E12	Trisomy 8 and 19, add2q, del6q	Imatinib	>5 m	R	(5)
2	43/M		E8a-E12	ND	Imatinib	>9 m	R	
3	61/M		E9-E12	ND	Imatinib	>8 m	R	
4	55/M		E10-E12	ND	Imatinib	NA	NA	
5	37/M	*BCR* (22q11)	E7-E12	ND	Allo-HSCT	85 m	R	(6)
6	3/M		E12-E12	ND	Allo-HSCT	50 d	D	
7	47/M		E1-E13	ND	Imatinib	14 d	R	(7)
8	57/M		E17-E12	ND	Imatinib	7 m	R	(8)
9	54/M	*KIF5B* (10p11)	E23-E12	ND	Imatinib	12 m	R	(9)
10	71/F	*CDK5RAP2* (9q33)	E13-E12	ND	Imatinib	3 m	R	(10)
11	64/M	*STRN* (2q24)	E6-E12	ND	Imatinib	24 m	R	(11)
12	51/M	*ETV6* (12p13)	E6-E12	ND	Imatinib	9 m	R	
13	44/M	*FOXP1* (3p13)	E23a-E12	ND	Imatinib	36 m	R	(12)
14	~60/F	*AKAP9* (7q21)	E23-E12	*RUNX1*, *DNMT3A*, and *WT1* mutation	Allo-HSCT	NA	D	(13)
15	27/M	*NRF1* (7q32)	E5-E12	ND	Imatinib	3 m	R	this case

ND, not detected; Allo-HSCT, allogeneic hematopoietic stem cell transplantation; R, remission; NA, not available; D, deceased.

The MLN-TK with cerebral infarction is rarely reported in the literature. Cerebral infarction caused by eosinophilia may involve three mechanisms: (i) endocardial damage leading to mural thrombosis, embolism, and infarction; (ii) a hypercoagulable state promoting thrombosis through eosinophil release of various proteins; and (iii) insufficient perfusion due to eosinophil-induced vascular permeability, microcirculation disturbance, and oxygen deficiency (23–25). Although imaging studies in this patient did not reveal any large vessel abnormalities, Cranial MRI identified a wedge-shaped hyperintense lesion at the junction of the right frontal, temporal, and parietal lobes, which is consistent with the typical characteristics of a watershed infarction. This finding may indicate localized ischemic changes, potentially resulting from small vessel inflammation or a state of hypoperfusion. Notably, after imatinib treatment, the hyperintense cortical signal in the right frontal-temporal-parietal junction area significantly reduced, indicating a potential association between the brain infarction and eosinophilia in this patient.

In summary, this is the first report to describe the *NRF1*::*PDGFRA* fusion gene in MLN-TK, which exhibits highly sensitivity to low-dose imatinib. Additionally, the patient in this case exhibited a rare occurrence of brain infarction associated with eosinophilia. This discovery not only highlights a novel genetic alteration in MLN-TK but also provides a basis for further investigation into the role of this fusion gene in disease pathogenesis and treatment responsiveness.

## Data Availability

The original contributions presented in the study are publicly available. This data can be found here: https://doi.org/10.6084/m9.figshare.28602737.
