# Supplementary material for: Identification of a novel NRF1::PDGFRA fusion in myeloid/lymphoid neoplasms with eosinophilia and tyrosine kinase gene fusions
Source: Front Oncol. 2025 Mar 25;15:1552928. doi: 10.3389/fonc.2025.1552928 (PMC11975939; doi:10.3389/fonc.2025.1552928)
Supplement: Supplementary file 1 [file DataSheet1.docx]

***Supplementary Material***

1 SUPPLEMENTARY FIGURE


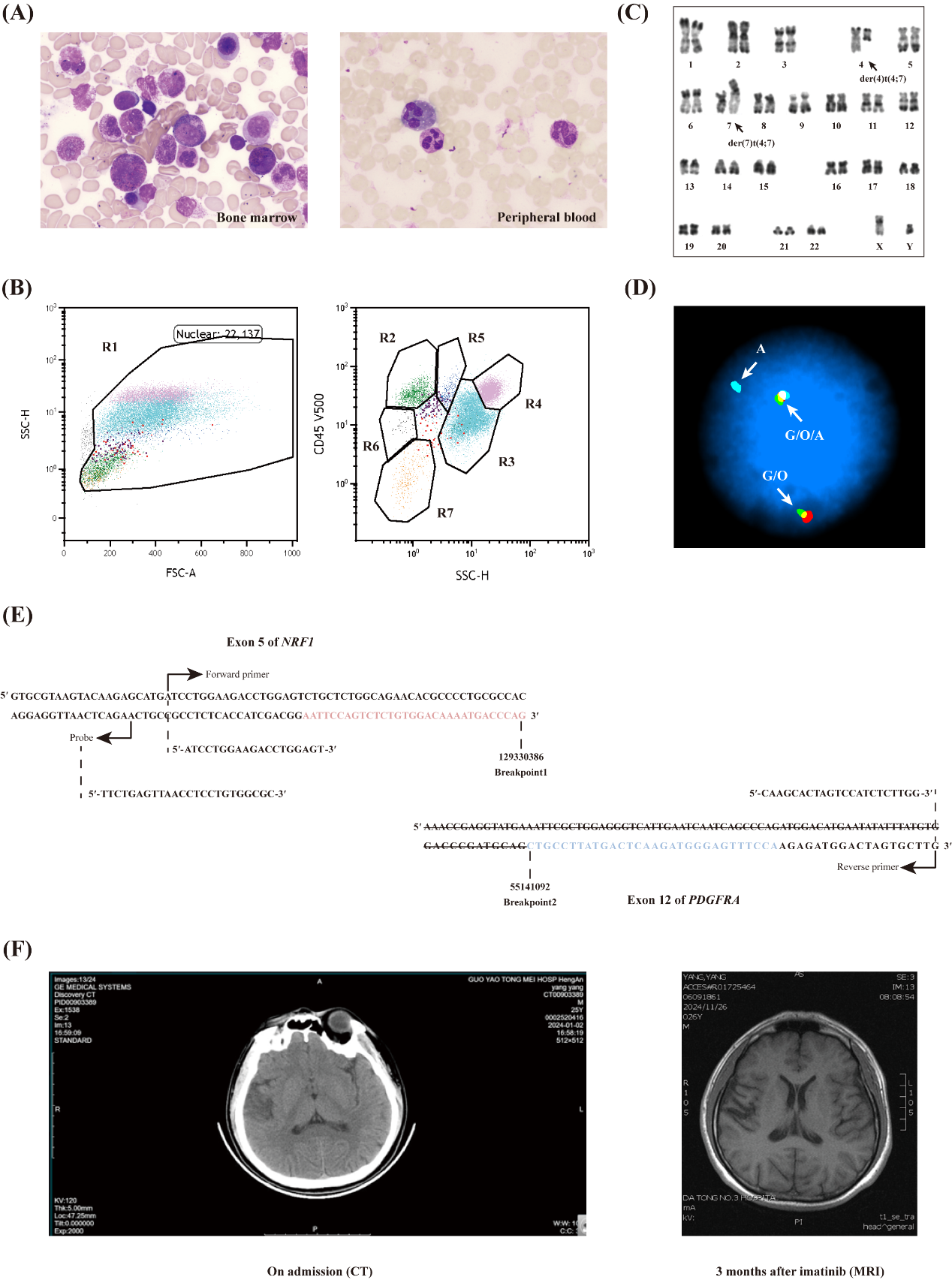


**Supplementary Figure 1.** Additional relevant analyses of patient specimens. (A) Morphological analyses of BM cells and peripheral blood cells show the significant granulocytic hyperplasia with increased cytoplasmic granules in the granulocytes, and the proportion of eosinophils was elevated, accompanied by vacuolar degeneration. (B) Flow cytometry analysis reveals a low proportion of myeloid and an increased proportion of eosinophils. R1: nucleated cell populations (98.38%); R2: mature lymphocyte populations (8.01%); R3: immature and mature granulocyte populations (55.81%); R4: eosinophil populations (29.4%); R5: mature monocyte populations (2.08%); R6: B progenitor cell populations (0.39%); R7: immature red blood cells (2%). (C) The karyotype showing abnormal chromosomes identified by R-banding. Abnormal chromosomes are marked with black arrows. (D) Interphase FISH hybridized with *PDGFRA* Tricolor Rearrangement Probe, which shows one overlapping green/orange/aqua signal (G/O/A), one overlapping green/orange signal (G/O), and one separate aqua signal (A). (E) Schematic illustration of the primers and probe binding sites on the cDNA of *NRF1*::*PDGFRA* designed for TaqMan RT-PCR analysis. The sequences of the forward primer, reverse primer, and probe are shown in the figure. (F) Cranial imaging before and after imatinib treatment.
